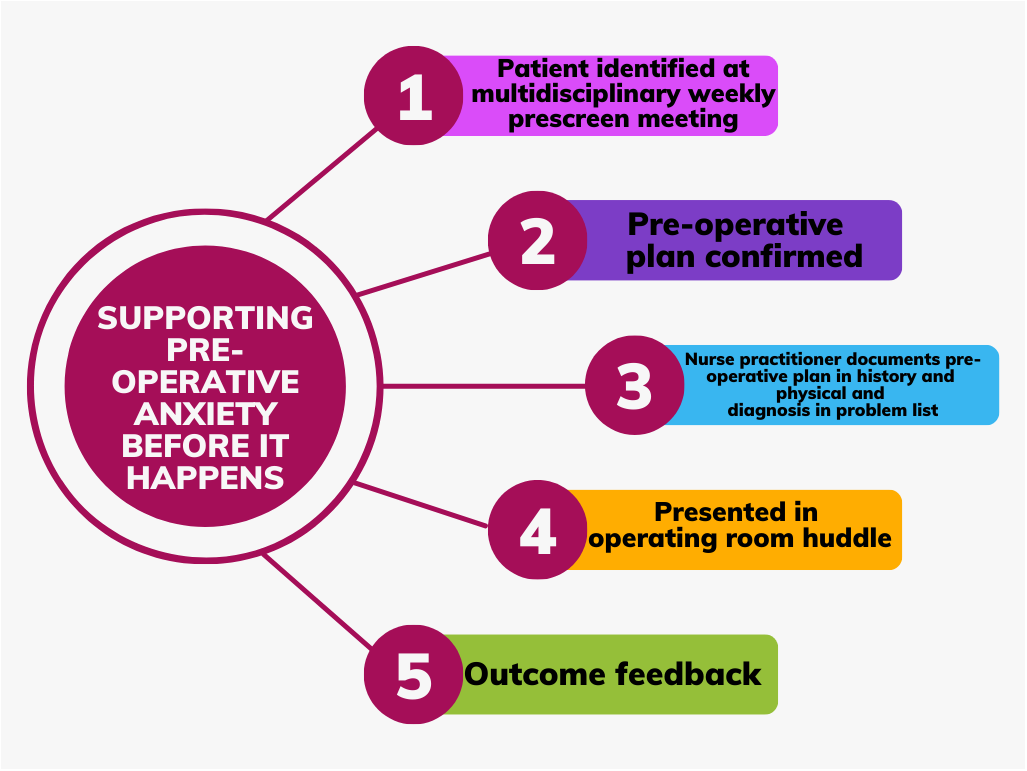# 676 Supporting Pre-Operative Anxiety Before It Happens

**DOI:** 10.1093/jbcr/iraf019.305

**Published:** 2025-04-01

**Authors:** Melissa Brown, Ellen O’Donnell, Tolga Ceranoglu, Kathy Prelack

**Affiliations:** Shriners Children’s - Boston; Shriners Children’s - Boston; Shriners Children’s - Boston; Shriners Children’s - Boston

## Abstract

**Introduction:**

Pre-operative anxiety (POA) in pediatric burn patients poses a challenge for recovery, as management of burn injuries often requires multiple surgical interventions over time. With an occurrence rate between 50-75%, early identification of POA is essential to prevent and mitigate long-term psychiatric and behavioral concerns. Upon evaluation, our facility process lacked a mechanism to identify anxious patients, develop treatment plans, communicate information or provide feedback on anxiety management. To address these concerns a uniform workflow was needed. This project was undertaken as a Quality Improvement Initiative and was not formally supervised by an Institutional Review Board.

**Methods:**

A multi-disciplinary workgroup evaluated processes and generated solutions. Four high-value touch points were identified offering opportunities for improvement in case management, child life, nurse practitioner and operating room (OR) roles. Key elements include prescreening, historical response to induction, anxiety-specific documentation, and inclusion of an OR huddle. (Figure 1) Optimized process was implemented on 04/01/24. Patients identified through prescreen process from 04/01/24-09/13/24 as having POA were included. Descriptive statistics and McNemar’s test for ordinal data were used to compare documentation of POA and if anxiolytics were offered between initial and most recent outpatient surgical encounters.

**Results:**

A total of 55 patients with a mean burn size of 28% total body surface area and age of 2.7 years were identified as having POA. About 78.5% were injured before age 5, with 59% of patients having acute admission over 4 weeks and an average of 16.13 surgeries. Of these, 51.8% were white/non-Hispanic, 35.7% Hispanic, 8.9% Asian, and 1.8% African American; 49.1% received initial care in another country, and 47.3% were non-English speakers. There was a significant increase in provision of pre-op anxiolytics between first and last outpatient surgical encounters (8 vs 41; p=0.001). Similarly, documentation of POA plan in the history and physical increased from 13 to 47 patient records (p = 0.001). POA listed in patient problem list also significantly increased (7 vs 18; p=0.005).

**Conclusions:**

Capturing patients’ POA improved post-implementation of new workflow. Multidisciplinary communication was a key component of success, but continued challenges exist. Reliable documentation within the medical record as well as engagement from all multidisciplinary members is crucial for efficiency.

**Applicability of Research to Practice:**

Further research including guardian perspective on POA intervention is necessary.

**Funding for the Study:**

N/A